# Plant-expressed bacteriophage lysins control pathogenic strains of *Clostridium perfringens*

**DOI:** 10.1038/s41598-018-28838-4

**Published:** 2018-07-12

**Authors:** Vaiva Kazanavičiūtė, Audrius Misiūnas, Yuri Gleba, Anatoli Giritch, Aušra Ražanskienė

**Affiliations:** 1 Nomads UAB, Geležinio vilko 29A, LT-01112 Vilnius, Lithuania; 2 Nomad Bioscience GmbH, Biozentrum Halle, Weinbergweg 22, D-06120 Halle (Saale), Germany

## Abstract

The anaerobic spore-forming bacterium *Clostridium perfringens* is a source of one of the most common food-borne illnesses in the United States and Europe. The costs associated with disease management are high and interventions are limited; therefore, effective and safe antimicrobials are needed to control food contamination by *C. perfringens*. A viable solution to this problem could be bacteriophage lysins used as food additives or food processing aids. Such antimicrobials could be produced cost-effectively and in ample supply in green plants. By using edible plant species as production hosts the need for expensive product purification can be reduced or obviated. We describe the first successful expression in plants of *C. perfringens*-specific bacteriophage lysins. We demonstrate that six lysins belonging to two different families (N-acetylmuramoyl-L-alanine amidase and glycosyl hydrolase 25) are active against a panel of enteropathogenic *C. perfringens* strains under salinity and acidity conditions relevant to food preparation environments. We also demonstrate that plant-expressed lysins prevent multiplication of *C. perfringens* on cooked meat matrices far better than nisin, the only currently approved bacteriocin food preservative to control this pathogen.

## Introduction

The anaerobic spore-forming bacterium *Clostridium perfringens* is a source of one of the most common food-borne illnesses in the United States, Europe and many other regions. *C. perfringens* gastroenteritis is caused by type A strains that produce the *C. perfringens* enterotoxin (cpe)^1^. The cpe-mediated food poisoning outbreaks typically are associated with temperature-abused meat and involve a large number of victims. Optimal conditions for food poisoning arise when food contaminated with cpe-positive *C. perfringens* spores is slowly chilled or held (or served) at a temperature range of 10–54 °C allowing germination and rapid growth of the pathogen^2,3^. Ingestion of viable cells leads to gastroenteritis.

The case-load and costs associated with this disease are high. For example, in the Netherlands the average incidence of *C. perfringens* gastroenteritis is estimated to be 171,000 cases per year, with total costs amounting to 23.2 million euros^4^. In the USA, an average annual incidence of more than 950,000 translates to a total disease management cost exceeding 342 million dollars^5^.

The adequate temperature control is of utmost importance for prevention of *C. perfringens* proliferation in food. However, it is not easily achieved in practice and inactivation of bacteria by additional means would be beneficial. Currently, there are very few technical options to inactivate bacteria in food. Most of the interventions involve heating or treating food with chemical preservatives (nitrate and nitrite, phosphate, organic acids) which can adversely modify the taste and quality of the products^6^. The polypeptide bacteriocin nisin is produced by *Lactococcus lactis* and is active against Gram-positive bacteria, including *Clostridium* spp. It is approved as a food preservative by the EU (E234), as well as by the World Health Organisation (WHO) and the US Food and Drug Administration (FDA). However, few studies have assessed the antimicrobial activity of nisin against *C. perfringens*^7–12^, and widespread outbreaks of *C. perfringens* gastroenteritis persist. Clearly, safe and effective new methods to control food contamination by *C. perfringens* are needed.

Recently, Schulz *et al*.^13^ described potential application of plant-produced colicins to control enterohemorragic *E. coli* in food. When produced in food species of plants, colicins gained ready marketing allowance in the USA as GRAS (Generally Recognized As Safe) antimicrobials to control *E. coli* in a wide range of food products (FDA GRN 000593, GRN 000676) as food processing aids. According to FDA CFR 21, Part 101, ‘food processing aids’ are substances that are added to a food for their technical or functional effect in the processing but are present in the finished food at insignificant levels and do not have any technical or functional effect in that food. As opposed to ‘food additives’, ‘food processing aids’ are exempt from labeling. Therefore, we explored a similar strategy for controlling *C. perfringens*, this time by evaluating plant-produced endolysins.

Bacteriophages have been used as natural control agents for bacterial infections since the early part of the 20^th^ century, mostly before the discovery and adoption of antibiotics. The growing antibiotic-resistance problem renewed interest in bacteriophages both as therapeutics and as bactericides in food processing. Several phage cocktails (e.g. Listex P100™, SalmoFresh™, ListShield™ and others) have been commercialized as GRAS (Generally Recognized as Safe in the USA) food processing aids, or for veterinary application (e.g. INT-401™) for controlling *C. perfringens* in poultry^14^.

Endolysins (lysins) are enzymes used by bacteriophages during the terminal stages of their replication cycle. The direct application of lysins offers the advantage of instant activity against susceptible bacterial targets. Antimicrobial activity of several *E. coli*–produced *C. perfringens* phage lysins Ply3626, PlyCP26F, PlyCP39O, psm, PlyCM and CP25L has been described^15–19^. We explored plant-based production of lysins because of the potential scalability and cost advantages of plant-based platforms^20^.

Recently we demonstrated the feasibility of producing bacteriophage lysins in plants by high-yield expression and purification of *Streptococcus pneumoniae*-targeting lysin Pal^21^. The activity of this plant-produced lysin was shown in bacteriolytic assays *in vitro* and its utility was demonstrated in a murine model of peritonitis. The success of the Pal lysin study encouraged us to proceed with the expression of *C. perfringens*-specific bacteriophage lysins. We first attempted production of lysins described in prior studies: Ply3626^15^, PlyCP26F, PlyCP39O^16^, psm^17^ and CP25L^19^. All lysins were efficiently expressed in plants with the sole exception of Ply3626, which expressed at very low level. In addition, from the phylogenetic tree of putative *C. perfringens* bacteriophage lysins created by Schmitz *et al*.^18^, we selected two lysins belonging to the GH25 protein family, ZP_02640173 (further in the text ZP173) and ZP_02640278 (further in the text ZP278). Both lysins were expressed at high level and demonstrated antimicrobial activity.

This study is the first to report successful expression of *C. perfringens*-specific lysins in plants. We demonstrate plant expression of lysins belonging to different families based on their mode of action (N-acetylmuramoyl-L-alanine amidase and glycosyl hydrolase 25). The lysins were shown active against a panel of *C. perfringens* serotypes associated with food-borne illnesses under salinity and acidity conditions that are relevant to food processing and preparation environments. We also demonstrate that plant-expressed lysins prevent multiplication of *C. perfringens* on cooked meat matrices better than nisin, the only currently approved bacteriocin food preservative for controlling this pathogen.

## Results and Discussion

### Identity of plant expressed lysins

The characteristics of all six plant–expressed endolysins are described in Table 1. As mentioned, three endolysins (CP25L, PlyCP26F and PlyCP39O) contain the N-acetylmuramoyl-L-alanine amidase at their catalytic domain, while psm contains a muramidase belonging to the Glyco_hydro_25 family^16,17,19,22^.

**Table 1:** Endolysins expressed in plants.

Lysin	Origin	Characteristics/mode of action	NCBI reference	Reference
psm	*Clostridium* phage phiSM101	GH25_Lyc-like and SH3b domain-containing protein	YP_699978.1	^17^
CP25L	*Clostridium* phage vB_CpeS-CP51	N-acetylmuramoyl-L-alanine amidase	YP_008058948.1	^19^
PlyCP26F	*Clostridium* phage phiCP26F	N-acetylmuramoyl-L-alanine amidase	YP_007004008.1	^16^
PlyCP39O	*Clostridium* phage phiCP39-O	N-acetylmuramoyl-L-alanine amidase	YP_002265435.1	^16^
ZP173	*C. perfringens* CPE str.4969, prophage region	GH25_LytC-like and SH3b domain-containing protein	WP_003469359	^18^
ZP278	*C. perfringens* CPE str.4969, prophage region	GH25_Lyc-like domain-containing protein	WP_003469445.1	^18^

Blast analysis of amino acid sequences of ZP173 (WP_003469359) and ZP278 (WP_003469445) revealed the GH25 domain indicating that both proteins are putative muramidases. Both proteins are annotated in GenBank as bacterial cell wall hydrolases. However, close inspection of *Clostridium perfringens* F4969 genome (contigs NZ_ABDX01000023.1 and NZ_ABDX01000024.1) reveals the presence of several phage genes in close proximity of both ZP173 and ZP278. ZP173 is flanked on its 3′ end with the gene coding for DNA adenine-specific methyltransferase, closely similar to that of *Clostridium* phage phiSM101. Several other phage protein-coding genes are also located in proximity: the protein homologous to gp17 of *Clostridium* phage phiS63, the phage minor structural protein, and the phage tail tape measure protein. ZP278 is also located in close proximity to phage–like protein coding genes: the phage minor structural protein, the tape measure protein, the capsid protein. Most likely, both ZP173 and ZP278 are localized in the remnants of the prophage regions entrapped in *C. perfringens* F4969 genome and belong to the bacteriophage lytic enzymes family.

**Figure 1:**
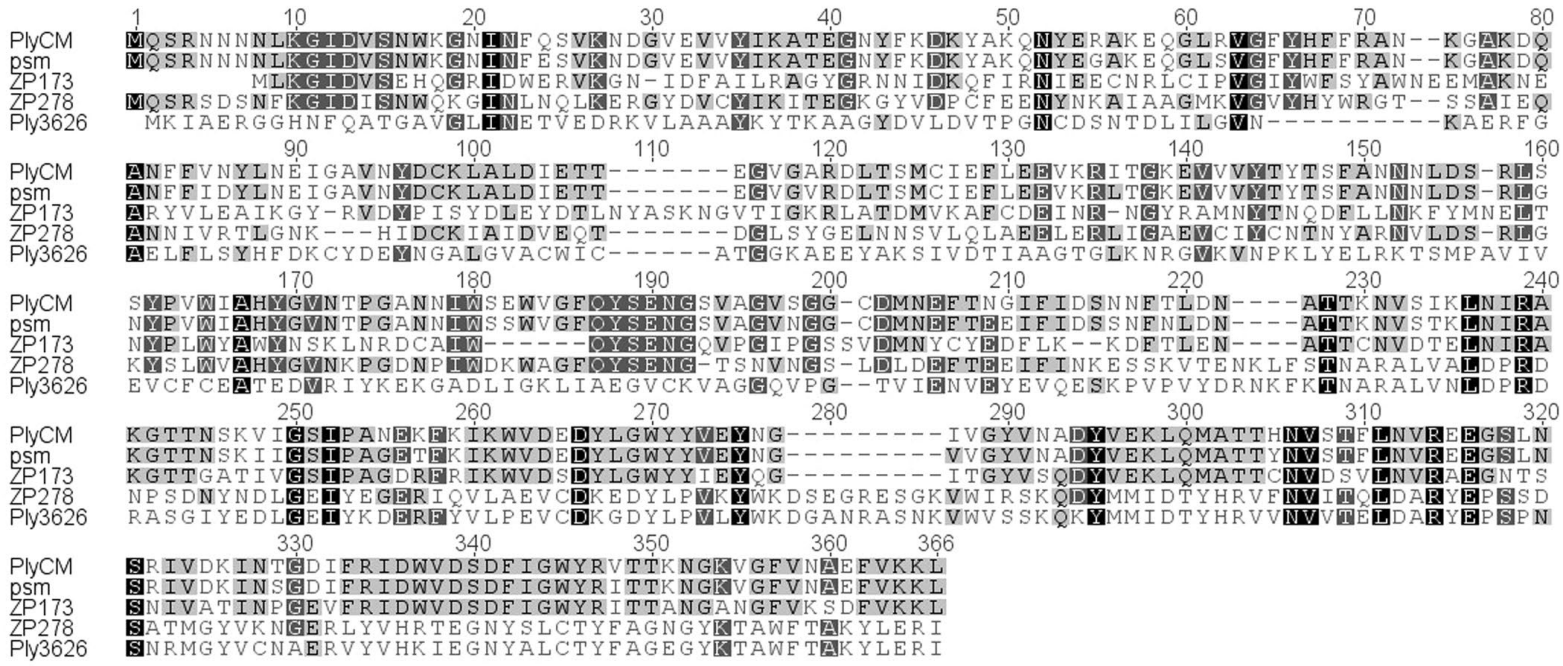
Alignment of PlyCM, psm, ZP173, ZP278 and Ply3626 amino acid sequences. Alignment of amino acid sequences was completed using Clustal W in Geneious and shading represents conserved identical residues shared among proteins.

ZP173 sequence alignment with other described *C. perfringens* lysins demonstrated closest similarity to psm (and its close relative PlyCM), with highest percentage of identity at its carboxy-terminal end (residues 177–335, 64.6% of identity), while the N-terminal end (residues 1–176) is only 29.7% identical to amino-terminal fragment of psm. ZP278 sequence alignment with other described *C. perfringens* lysins demonstrated closest similarity to psm (amino-terminal end, including the catalytic domain (residues 1–179, identity 47.2%), and to Ply3626 C-terminal fragment (residues 211–351, 74.5% of identity). Amino acid sequence alignments of PlyCM, psm, ZP173, ZP278 and Ply3626 are presented in Fig. 1.

### Lysin expression in plants, purification and characterization

**Figure 2:**
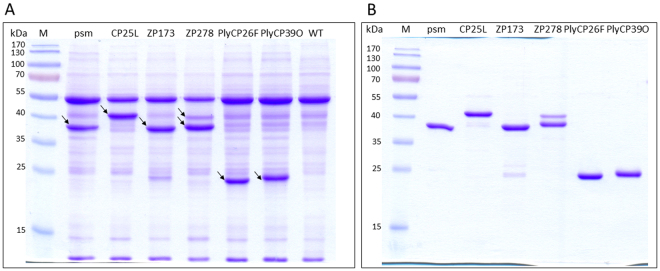
Expression of *Clostridium perfringens* bacteriophage lysins. Coomasie stained SDS-PAGE of crude plant extracts (**A**) and purified lysins (**B**). (**A**) Plant material (50 mg) was harvested at 5 or 7 days post spraying (psm, CP25L, ZP173, ZP278) or post infiltration (PlyCP26F, PlyCP39O), ground in liquid nitrogen and extracted with 50 mM sodium phosphate, 5 mM DTT, 150 mM NaCl, (pH 7.5). 4 µl of plant extract was resolved in 12.5% polyacrylamide gel for Coomassie staining. M – PageRuler Prestained protein ladder (Thermo Fisher Scientific Baltics), psm, CP25L, ZP173, ZP278, PlyCP26F, PlyCP39O – extracts of *N. benthamiana* leaves, transfected with lysins expression constructs, WT – crude extract of non - sprayed *N. benthamiana* leaves. Bands corresponding to recombinant lysins are marked by arrows. (**B**) Lysins were purified by two-step chromatography as described in Purification section of Methods and Suppl. Text S1, and resolved in 12.5% polyacrylamide gel for Coomassie staining.

*N. benthamiana* leaves infiltrated or sprayed with *A. tumefaciens* strains carrying vectors of interest were harvested and analysed for protein expression. Specific bands of expected size were detected in Coomassie-stained SDS-PAGE gels for psm, ZP173, PlyCP26F, PlyCP39O and CP25L lysins (psm and ZP173 ∼38-39 kDa, ZP278 ∼40 kDa, PlyCP26F and PlyCP39O ∼24 kDa, CP25L ∼43 kDa) (Fig. 2A). ZP278 was detected as a double band; the upper band corresponded to predicted MW of 40.2 kDa, whereas MW of the main band was smaller by about 2 kD. Such relative mobility in the gel is probably related to the cysteine-rich nature of this protein; re-oxidation of cysteine residues might occur during denaturation leading to the formation of intramolecular disulphide bridges.

All six lysins were expressed in plants very efficiently, and simple visual inspection suggested that they comprise at least 30 percent of total soluble leaf protein. All lysins could be extracted in soluble form by using one single extraction buffer (Fig. 2A). Expression of lysins in spinach also gave similar results (Suppl. Fig. S1).

We anticipate that purification to homogeneity of lysins produced in edible plant species would be not compulsory for food-related applications, and that only partial purification of lysins would be sufficient. We base this conclusion on our prior regulatory experience with *E. coli* colicins^13^; GRN 593. However, in these studies we decided to use purified lysins to characterize these proteins and to ascertain unambiguously the potency of plant-produced lysins to eradicate *C. perfringens in vitro* and in a model food system.

We purified all six lysins to homogeneity by protein chromatography, using the general scheme: homogenization of sample, clarification, HIC chromatography, followed by desalting and CEXC or AEXC column (Suppl. Text S1, Suppl. Fig. S2 and Fig. 2B). The average yields of purified proteins determined from several purifications were as follows: 550 µg/g of fresh leaf weight for ZP173, 1150 µg/g for ZP278, 300 µg/g for CP25L and psm, and 150 µg/g for PlyCP26F and PlyCP39O.

Four purified lysins (psm, CP25L, ZP173 and ZP278) were subjected to mass spectrometric analysis in order to confirm that they are identical to bacteriophage proteins and have intact N- and C- termini. Molecular mass analysis, in-source decay (ISD) analysis and T3-sequencing confirmed that all four lysins have intact protein termini. Molecular mass analysis confirmed that the detected molecular masses of all four lysins correspond very closely to the predicted theoretical masses. No post-translational modifications were detected for CP25L, while the remaining three lysins (psm, ZP173 and ZP278) are acetylated at their N- terminus (Suppl. Text S2, Suppl. Table S1, Suppl. Figs S3,S5).

Thus, after successfully expressing *S. pneumoniae*-targeting bacteriophage lysin Pal^21^, we were also able to achieve high-yield expression of six *C. perfringens* bacteriophage lysins, demonstrating again the suitability and versatility of our plant-based transient expression system for producing these antimicrobial proteins.

### Activity of plant-made lysins against *C. perfringens in vitro*

**Figure 3:**
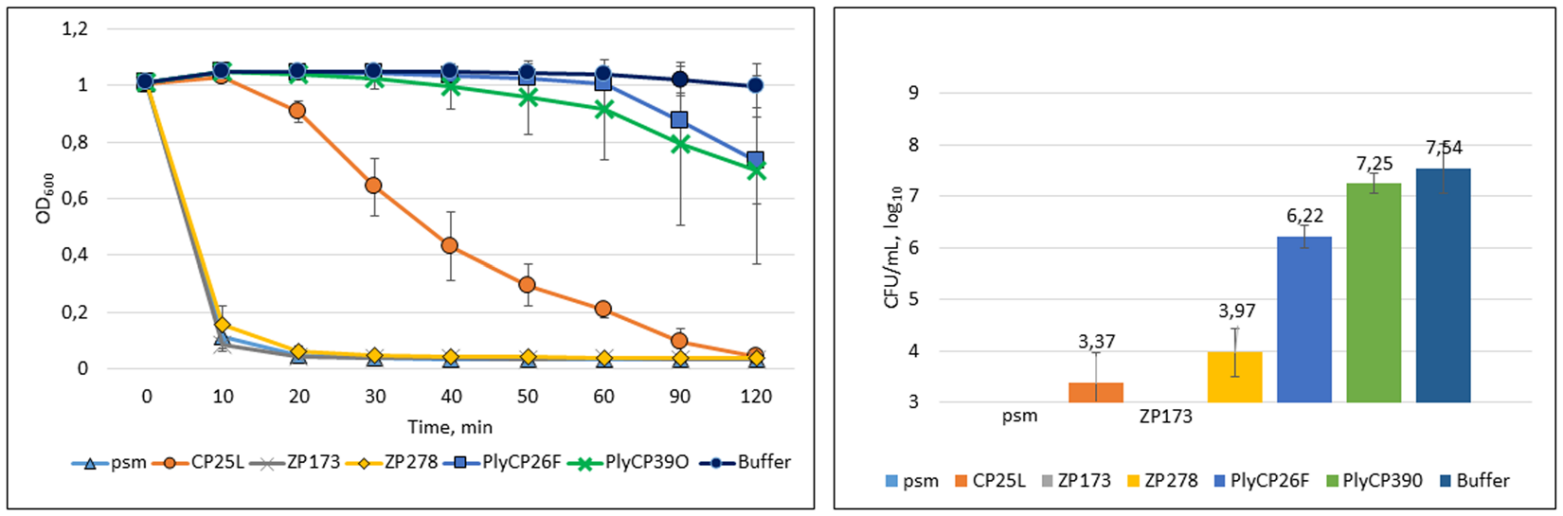
Bacteriolytic activity of *N. benthamiana*–expressed lysins against *C. perfringens* NCTC8237. *C. perfringens* NCTC8237 was suspended in citrate-phosphate buffer supplemented with 50 mM NaCl, pH 5.5 and treated with 10 µg/ml of lysin. Left panel: the turbidity reduction of the bacterial suspension after incubation with lysins. Right panel: *C. perfringens* cfu counts after 60 min. of co-incubation with lysins. Data are the mean ± SD of three independent experiments.

**Figure 4:**
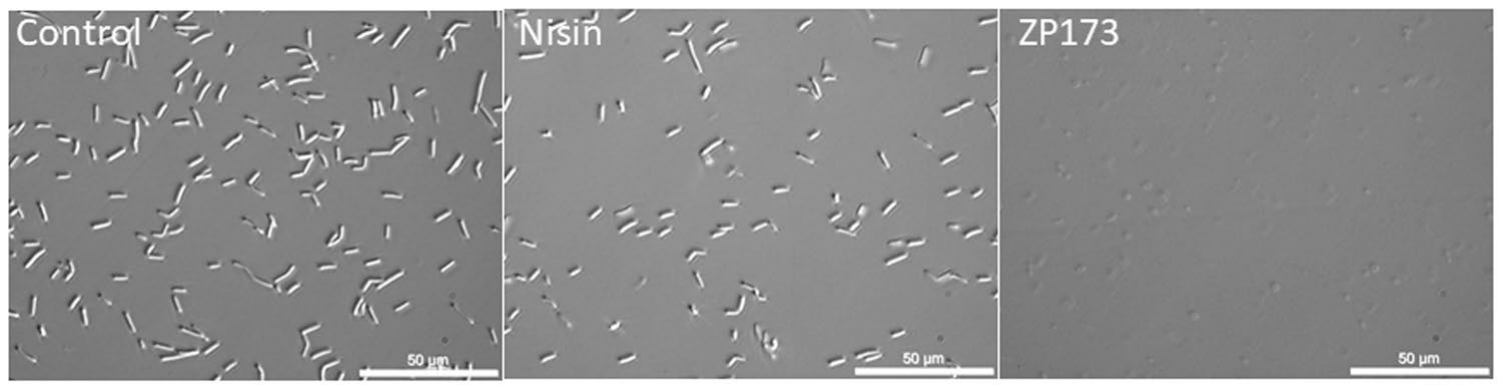
The morphology of untreated, ZP173-treated and nisin-treated NCTC8237. *C. perfringens* was incubated with 5.5 µg/ml of nisin or with 5 µg/ml of ZP173 in citrate-phosphate buffer with 50 mM NaCl (pH 5.5). The microscopy images (x1,000 magnification, phase contrast) were taken after 90 min of incubation at RT.

Plant expressed *C. perfringens* phage lysins psm, ZP173, ZP278, CP25L, PlyCP26F and PlyCP39O were at first tested with *C. perfringens* type strain NCTC8237 (Fig. 3). Incubation of bacterial suspension with lysins constantly resulted in complete clarification of suspension for ZP173, psm, ZP278 and CP25L. Phase contrast microscopy of lysin-treated bacteria suspensions confirmed that, within 90 minutes, bacteria were completely lysed by the action of either of these lysins, while they remained intact in control samples and also in samples, treated by nisin. The representative picture is shown (Fig. 4). OD_600_ reduction was very rapid for psm, ZP173 and ZP278 (suspension almost clarified after 10 min and baseline reached after 20 min) and slower for CP25L (baseline reached after 2 hours). PlyCP26F and PlyCP39O were able to clarify bacterial suspension only partially, and acted more slowly, with OD_600_ starting to decline after 40–50 min of incubation.

The CFU counts after 1 hour of incubation correlated well with OD measurement data. The psm and ZP173 treatments reduced *C. perfringens* cfu/ml number below the limit of detection (by more than 4.5 logs), CP25L treatment reduced cfu/ml number by 4.2 log_10_, ZP278 by 3.6 log_10_, PlyCP26F by 1.3 log_10_ and PlyCP39O by 0.3 log_10_ (10 µg/ml, corresponding to 115–130 nM, depending on the lysin). Thus, five lysins were shown to be functional in killing the *C. perfringens* type strain NCTC8237, with PlyCP39O demonstrating only statistically insignificant activity.

### Activity of lysins under salinity and acidity conditions commonly found in food

*C. perfringens* most commonly causes gastrointestinal infections after ingestion of cooked yet temperature-abused meat products and catered food^2,3^. For the successful use of lysins as food antimicrobials, they must be active under pH and salinity conditions usually found in food processing and/or preparation. Hence, we evaluated the influence of NaCl concentration, pH and temperature on lysins’ antibacterial activity.

#### Salinity

**Figure 5:**
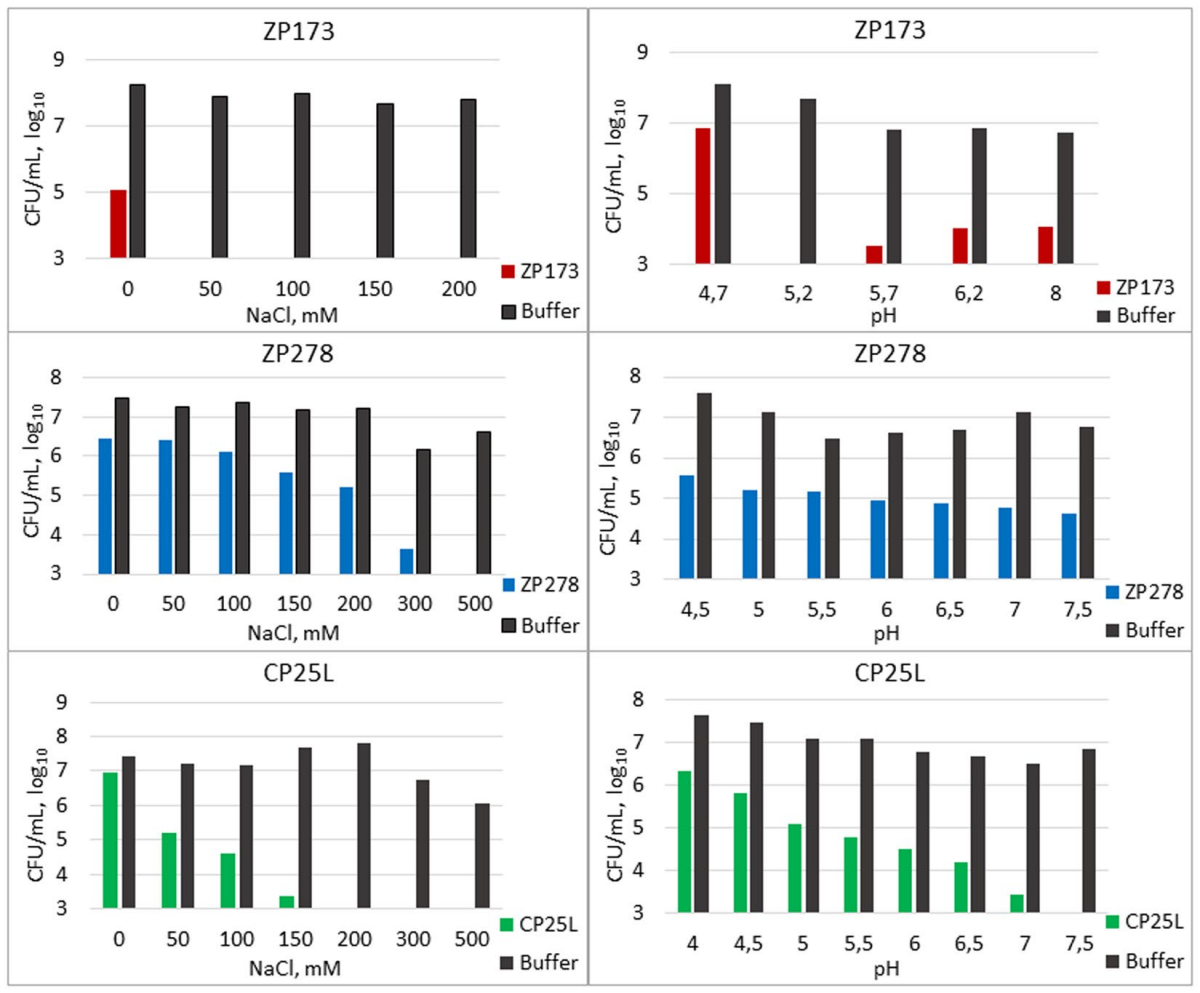
Activity of plant – expressed lysins at different concentration of NaCl and at different pH. Left panel, NaCl. *C. perfringens* NCT8237 was grown in TSB under anaerobic conditions to OD_600_ = 0.6-0.7, centrifuged and suspended in citrate-phosphate buffer of pH 5.5 with different NaCl concentrations. 1 ml of bacterial suspension was mixed with 3 µg of purified ZP173, 34 µg of ZP278 or 7.5 µg of CP25L and incubated at RT for 60 min. Right panel, pH. *C. perfringens* NCT8237 was suspended in citrate-phosphate buffer with 100 mM NaCl (buffer pH from 4.5 to 8.0). 1 ml of bacterial suspension was mixed with 4 µg of ZP173, 27 µg of ZP278 or 5 µg of CP25L and incubated at RT for 1 h.

Ready-to-eat meat products usually contain various amounts of added salt (NaCl). In cured or cooked meat products (e.g. ham, sausages) NaCl concentration can be as high as 150–350 mM. We evaluated the influence of 50 mM–500 mM NaCl concentration on the antibacterial activity of plant-produced lysins. An increasing concentration of salt positively influenced the activity of all three tested lysins, ZP173, ZP278 and CP25L (Fig. 5, left). As little as 50 mM of NaCl was sufficient to achieve maximum activity of ZP173 at the concentration tested. The activity of CP25L also increased with increasing NaCl concentration up to 200 mM, and no bacterial colonies were detected at higher concentrations. The activity of ZP278 continued to increase up to 500 mM (the highest concentration tested). These results are encouraging as they demonstrate that the antibacterial effect of lysins is highly compatible with the salt content found in various types of food. We did not evaluate the influence of salinity on the activity of psm, as it was already described that this lysin is most active at NaCl concentration of 250 mM^17^. In contrast, ZP173 exhibited high activity even in buffer without NaCl, reducing CFU count by more than three orders of magnitude, and could possibly be used in foods with low salt content. For example, the NaCl concentration in raw meat and fish is approximately 10 mM, although it can be several-fold higher in some sea foods^23^.

#### pH

Most meat-based foods are slightly acidic, ranging from pH 5.0 to 7.0, in part naturally, and in part because organic acids are added to the processed product for better preservation. We evaluated the activity of lysins at pH values ranging from 4.0 to 8.0 and obtained quite different results for the three lysins (Fig. 5, right). The activity of ZP173 was highest at pH 5.2 and decreased towards lower and slightly towards higher acidity. The acidity of test buffer had little influence on the activity of ZP278. Conversely, pH strongly influenced the activity of CP25L; it consistently increased up to the highest level tested, pH 7.5.

As already described, psm showed optimal activity at pH 6.5–7.0, and the optimal activities were 6.8 and 8.2 for PlyCP26F and PlyCP39O, respectively^16,17^. Considering that both raw meat and processed meat products have pH values below 7.0, the activity of CP25L and PlyCP39O, in particular, would be expected to be suboptimal in such foods.

#### Temperature

Food additives should prevent multiplication of bacteria when food is stored under improper conditions. *C. perfringens* does not multiply in properly refrigerated food. The lower growth limit for *C. perfringens* is 6 °C^24^. In this study, we examined the activity of lysins after a long-term storage at 4 °C, and their stability and activity in warmer environments ranging from RT (room temperature) to elevated temperature conditions (37 °C). Our experimental design aimed at modeling food that had been kept refrigerated, then taken out and kept out of refrigeration for indeterminate time.

**Figure 6:**
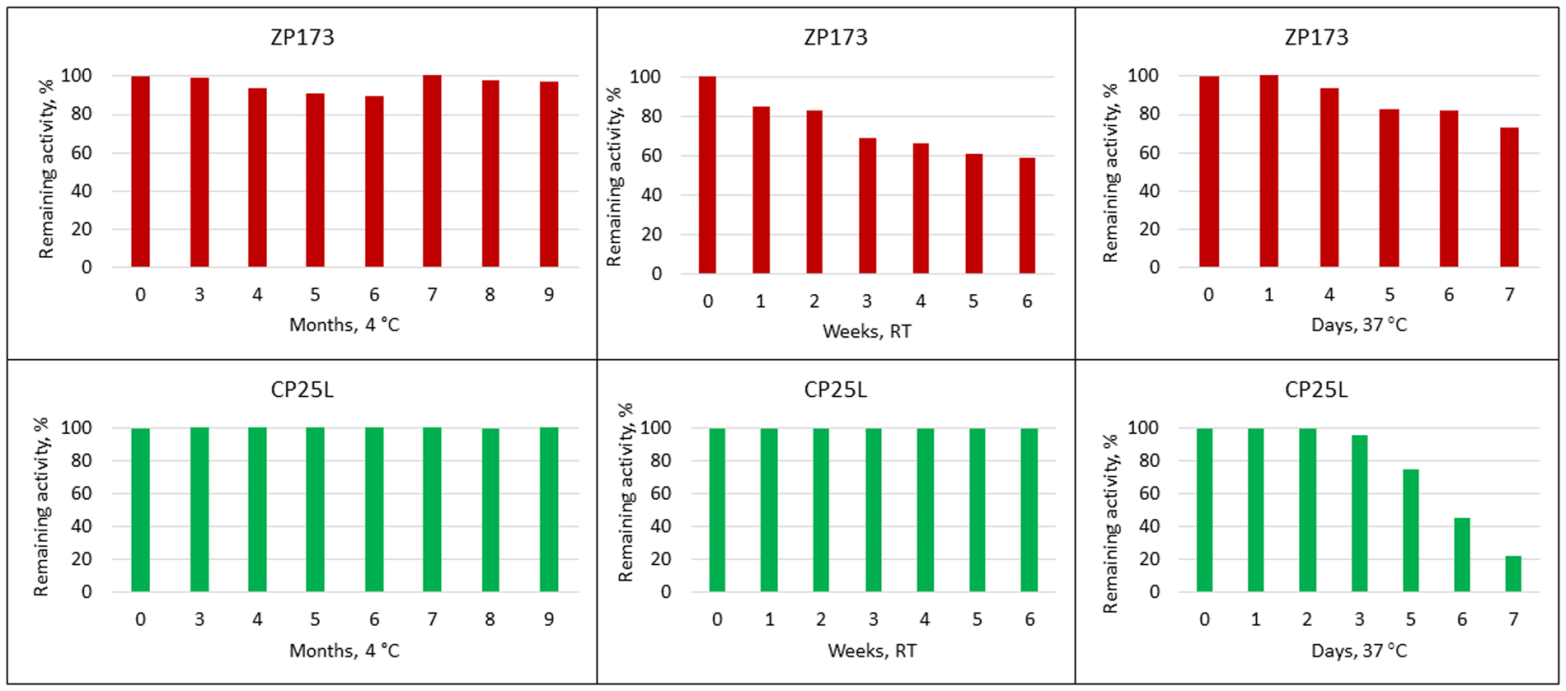
Remaining activity (%) of lysins after incubation at 4 °C, RT and 37 °C. Left panel: the purified lyophilized lysins were dissolved in milli-Q water and stored at 4 °C for up to 9 months. For activity assays, *C. perfringens* NCTC8237 was grown to OD_600_ = 0.8 in TSB anaerobically. Following centrifugation, bacteria were suspended in citrate-phosphate buffer with 50 mM NaCl, pH 5.5. 1 ml of bacteria was combined with 2 µg of purified lysin and incubated at RT for 1 hour. Middle panel: The lysins activity after long-term storage at room temperature. The purified lysins were incubated at RT for 1 to 6 weeks. *C. perfringens* NCT8237 was suspended in 1xPBS, pH 7.3 (CP25L) or citrate-phosphate buffer with 100 mM NaCl, pH 5.5 (ZP173). Bacterial suspension was mixed with 2 µg/ml of ZP173 or CP25L and incubated at RT for 1 hour. Right panel: the purified lysins were incubated at 37 °C for 1 to 7 days. *C. perfringens* NCT8237 was suspended in PBS, pH 7.3, mixed with 2 µg/ml of ZP173 or CP25L and incubated at RT for 1 hour. Remaining activity percent was calculated by taking activity of freshly solubilized lysins (from purified lyophilized stock) Δlog_10_ CFU/mL value as 100%.

Two lysins, ZP173 and CP25L, were evaluated. We detected no decline in activity of either lysin after storage for up to 9 months at 4 °C (Fig. 6). CP25L was also extremely stable at room temperature, and its activity remained unchanged after six weeks of storage. At 37 °C the activity of CP25L remained stable for two days, and then gradually decayed after 3 days, retaining about 20% of its original activity after one week. ZP173 was also relatively stable at RT and retained 60% of its original activity after six weeks of storage. ZP173 had superior stability to CP25L at 37 °C, conserving more than 70% of its original activity after one week of storage.

The thermal stability of his-tagged *E. coli*-expressed CP25L had been evaluated by Gervasi *et al*.^19^. In the described study, CP25L was equaly stable at 4 °C, but began losing activity at room temperature after 15 days of storage; hence, plant-produced CP25L exhibits better stability than its bacterially produced counterpart.

We conclude that purified ZP173 and CP25L lysins are extremely stable at 4 °C, retain high activity after several days and weeks of storage at ambient temperature, and exhibit significant functionality even after several days at 37 °C.

### Activity of plant-made lysins against *C. perfringens* food-contaminating strains

**Figure 7:**
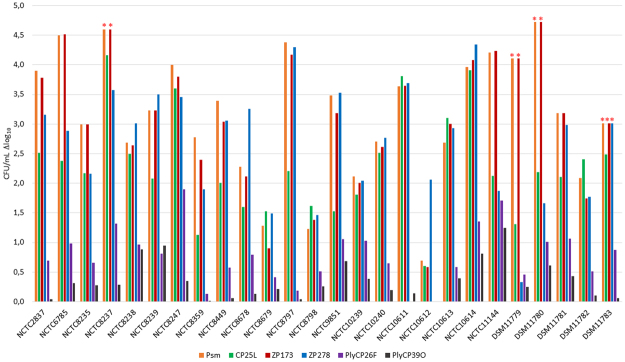
Killing of *C. perfringens* food strains by *N. benthamiana* – expressed lysins. CFU/mL Δlog_10_ of different *C. perfringens* strains treated with each lysin separately. *C. perfringens* strains were grown in TSB under anaerobic conditions to OD_600_ appr. 0.8 and suspended in citrate-phosphate buffer, 50 mM NaCl, pH 5.5. Lysins were added to bacterial suspension at final concentration of 10 µg/ml. Serial dilutions for cfu counts were done in PBS, pH 7.3 after 60 min. of co-incubation with lysins. *- no colonies were detected on plates.

To better characterize the range of antibacterial activity, we screened all six lysins against a collection of 26 *C. perfringens* Type A strains isolated mostly from food or from human feces after documented cases of food poisoning (Suppl. Table S2). All 26 strains were sensitive to treatment with one or more lysins at the concentration used (10 µg/ml = 10 ppm). Different strains demonstrated different susceptibility patterns, but in general, treatment with psm, CP25L, ZP173 and ZP278 reduced cfu counts most dramatically, while PlyCP26F and PlyCP39O were less effective (Fig. 7). Eradication of bacteria bellow the limit of detection was achieved for some strains by psm, ZP173 and ZP278 treatment (Fig. 7, marked by asterisks). Psm and ZP173 caused the most dramatic reduction in cfu counts, reaching a net difference of >4 logs for 7 and 8 strains, respectively. Four lysins (psm, CP25L, ZP173 and ZP278) were able to reduce cfu counts of 14 to 17 strains by 2 to 4 logs. For PlyCP26F and PlyCP39O, the maximum cfu reduction achieved was in the range of 0.5–2 logs. PlyCP26F could reduce cfu counts to that extent for 20 strains, and PlyCP39O for 6 strains only (Fig. 7).

The great variation of lytic activity against different strains exerted by plant-produced lysins is not surprising, as it has been previously reported for *C. perfringens* bacteriophage lysins psm, CP25L, PlyCP26F, PlyCP39O and PlyCM^16–19^. However, no previous studies exist where the lytic activity of several lysins was compared using the same panel of *C. perfringens* food-relevant strains. Our results suggest that although some strains (NCTC8679, NCTC8798) appear more recalcitrant than others to treatment by all lysins tested, in general all strains were susceptible to lysin activity albeit with differences in strain-to strain susceptibility. Among the six tested lysins, only psm and ZP173 demonstrated very similar patterns of activity, most likely because of high homology of both proteins in their C-terminal cell wall binding domains. PlyCP26F and PlyCP39O demonstrated relatively low anti-clostridial activity in this experiment. The probable cause might be that the pH conditions used in our study (pH 5.5) were suboptimal for both lysins. As described, at pH 5.5 PlyCP26F demonstrated only 30% and PlyCP39O only 20% of their maximal activity^16^. However, our aim was to examine how *C. perfringens* could be eradicated under conditions similar to those found in food processing and/or preparation environments; thus, our justification for evaluating lysins at slightly acidic pH values found in many contamination-susceptible foods.

Based on these results, *C. perfringens* eradication could be more successfully achieved by simultaneous treatment with several lysins (i.e. a mixture) rather than by treatment with individual lysins. For example, three plant-produced lysins (psm or ZP173, ZP278 and CP25L) could be used to control the broadest spectrum of *C. perfringens* food-relevant strains.

In addition, as these lysins represent enzymes of different families, a synergistic effect of muramidases and alanine amidases could be achieved by using combinations of lysins, as has been described for *Streptococcus* phage lysins^25,26^. However, we did not assess such synergistic effects with *C. perfringens* phage lysins.

### Activity of plant-made lysins against *C. perfringens* on cooked meat matrices

After demonstrating activity of plant-produced lysins *in vitro*, we next analyzed whether lysins are equaly able to decrease the number of viable vegetative *C. perfringens* cells on food matrices. To simulate conditions of improperly kept food, we used minced cooked turkey at room temperature inoculated with vegetative *C. perfringens* cells at 10^4^ cfu/ml. Currently, the polypeptide nisin is the only bacteriocin approved as a food preservative, which explains its increasingly common use by the food industry. Nisin has antimicrobial activity against Gram-positive bacteria and in particular against spore-forming species, and is able to inhibit both vegetative cells and the outgrowth of germinating spores^27^. However, although nisin showed inhibitory effect against spore outgrowth and vegetative cells of *C. perfringens* in laboratory conditions, no inhibitory effect of nisin was observed against *C. perfringens* spores inoculated in a meat model system^11^, suggesting that nisin only arrests the outgrowth of germinated spores. Nisin action against vegetative cells can either be bactericidal or bacteriostatic depending on the concentration of nisin, concentration of bacteria, bacterial strain, physiological state and exposure conditions, and as reviewed in^12^, in the majority of cases nisin’s action is sporostatic rather than sporicidal.

**Figure 8:**
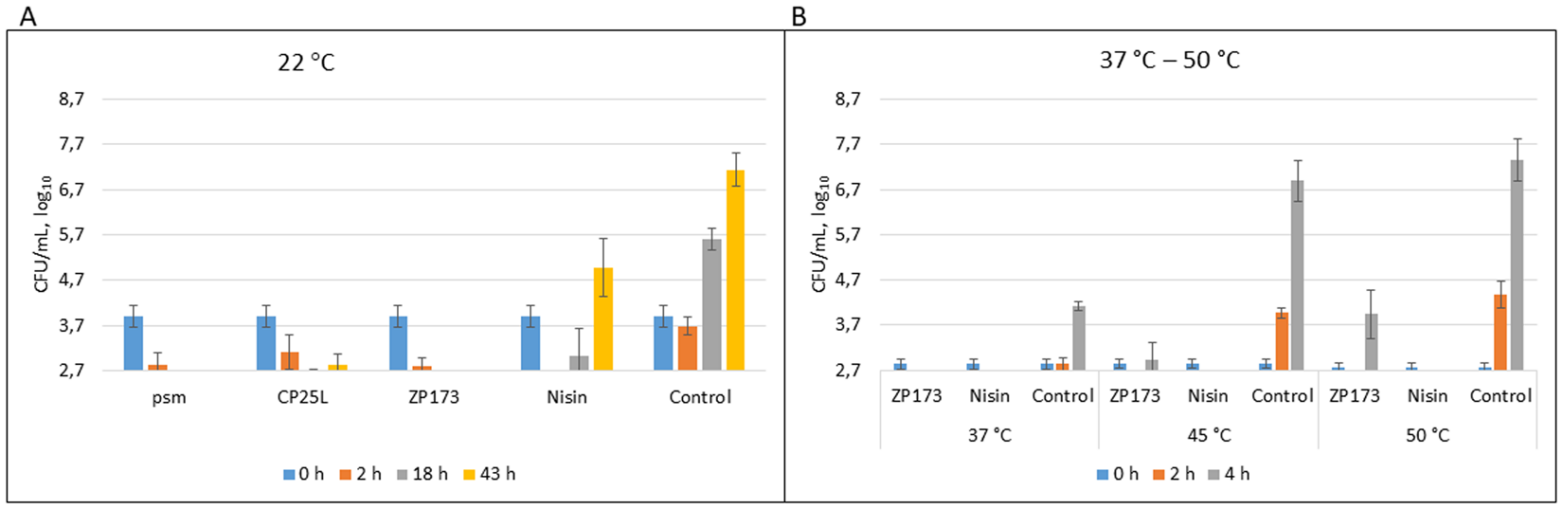
Activity of purified lysins in *C. perfringens*-contaminated turkey. (**A**) Activity of purified lysins in *C. perfringens*-contaminated turkey at room temperature. *C. perfringens* NCTC8237 bacteria were mixed with 10 g of minced cooked turkey meat at 4 log_10_ cfu/ml, 3 ml of citrate-phosphate buffer supplemented with NaCl (final concentration of 1.5%), pH 5.5 and 2.5 µg/ml of purified lysin or 5 µg/ml of nisin. 0 h – cfu counts of samples before addition of lysins or nisin. 2 h, 18 h and 43 h – cfu counts of samples, incubated at RT anaerobically for the indicated time. Data are the mean ± SD of four independent experiments. (**B**) Activity of ZP173 and nisin in *C. perfringens*-contaminated turkey at 37 °C–50 °C. *C. perfringens* NCTC8237 bacteria were mixed with 10 g of minced cooked turkey meat at 3 log_10_ cfu/ml, 3 ml of citrate-phosphate buffer with NaCl (final concentration of 1.5%), pH 5.5 and 5 µg/ml of purified lysin or 5 µg/ml of nisin and incubated at 37 °C, 45 °C and 50 °C anaerobically. 0 h – cfu counts of samples before addition of lysins or nisin. 2 h and 4 h – cfu counts of samples, incubated for the indicated time. Data are the mean ± SD of three independent experiments.

In our experimental conditions, no bacteria following 5 µg/ml nisin treatment were detected after 2 hours, indicating that nisin is active against *C. perfringens* (Fig. 8A). However, low titers of bacteria resurfaced after 18 hours and at 43 hours bacterial titers reached almost 5 log_10_ cfu. Thus, although nisin retarded multiplication of *C. perfringens* at the concentration used (which is nearly the maximal concentration allowed for its use as a food additive) it could not eradicate bacteria completely. In sharp contrast, two of the tested lysins, psm and ZP173, used at a two-fold lower (weight to weight) concentration than nisin (2.5 µg/ml), or about 25-times lower molar concentration, completely eradicated bacteria and no colonies were detected after 18 or 43 hours of incubation. CP25L was also very efficient and after 43 hours of incubation bacterial titers were reduced by 4 log_10_ cfu compared to control (Fig. 8A).

Thus, under the experimental conditions used, all three plant-expressed lysins efficiently protected cooked meat from *C. perfringens* spoilage, providing better protection than nisin. We repeated this experiment with the mix of randomly chosen five *C. perfringens* FP strains and lysins at concentration of 5 µg/ml. In this experiment, all antimicrobials tested, nisin and lysins, eradicated bacteria to the threshold of detection or to only slightly above it after 2 and 4 hours of incubation. However, only CP25L was able to stop proliferation of bacteria after 43 hours of incubation (Suppl. Fig. S6).

We further investigated the possibility that lysins are able to prevent or delay *C. perfringens* proliferation in contaminated food at higher temperatures, 37 °C, 45 °C and 50 °C. ZP173 was used in this experiment as one of the best performing lysins. Although *C. perfringens* grows very fast in elevated temperatures, both ZP173 and nisin effectively prevented proliferation of bacteria up to 4 hours at all incubation temperatures (Fig. 8B). This allows the suggestion that lysins could be used to prevent *C. perfringens* proliferation not only for food that is normally kept refrigerated (ex. cold meats), but also in a hot catered food (ex. soups, stews, gravies) which is frequently causing *C. perfringens* food infections as it is prepared beforehand and kept warm for extended periods of time^2,3^.

We conclude that plant-expressed lysins show utility as candidate food-protection antibacterials against *C. perfringens* when evaluated under conditions that realistically model food processing, preparation and serving scenarios.

Plant virus-based expression systems, in which the foreign mRNA encoding a protein of interest is amplified by the replicating virus, can produce very high levels of proteins in leaves and other tissues^28^. Furthermore, plant-based expression systems using Generally Recognized as Safe (GRAS) hosts (food species) have clear advantages when producing proteins for which extensive purification may need to be avoided. Recently, we used this system to produce in plants several colicins and pyocins^13,29^. Plant-made colicins have received marketing allowance through the US Food and Drug Administration’s (FDA) GRAS regulatory review process. Like colicins, bacteriophage lysins comply with GRAS criteria and could be used for treatment of foods and as human and animal therapeutic alternatives to antibiotics. It is anticipated that *C. perfringens* bacteriophage lysins produced in edible plants could be safely used as food additives or processing aids even if only partially pure. Thus, despite successful expression of *C. perfringens* bacteriophage lysins in *E. coli*, plant-based expression might be advantageous when the lysins are intended for food safety interventions.

## Methods

### Bacterial strains and growth conditions

*Agrobacterium tumefaciens* strains used in this study are listed in Suppl. Table S3. *Escherichia coli* strain DH5α was used as a recipient for all cloning procedures. Both *E. coli* and *A. tumefaciens* cells were grown in LB medium at 37 °C or 30 °C, respectively. All media were supplemented, when necessary, with 100 μg/ml ampicillin, 50 μg/ml spectinomycin, 50 μg/ml kanamycin and 50 μg/ml rifampicin. *C. perfringens* cultures were grown in TSB medium at 37 °C in anaerobic conditions using AnaeroGen gas generating system (Oxoid, UK). *C. perfringens* strains used in the study are listed in Suppl. Table S2.

### Construction of expression vectors

Plant – optimized gene coding sequences were synthetized by Eurofins, Austria (psm, PlyCP26F, PlyCP39O) and GenScript, USA (CP25L, ZP173, ZP278). All sequences have been deposited in the NCBI database in GenBank with the accession numbers MH509972 to MH509977.

The sequences were inserted as BsaI-BsaI fragments into the pICH31070 Δ lacZ plasmid (MagnICON deconstructed tobacco mosaic virus (TMV) system 3′ provector^30^ and into the pICH29912 (assembled TMV-based MagnICON vector for cytosolic expression^31^). Obtained plasmids were used to transform *A. tumefaciens* GV3101. *A. tumefaciens* strain containing pICH1401 with expression cassette of *Streptomyces* phage C31 integrase was used for co – infiltration with 5′ and 3′ provectors.

The T-DNA regions of obtained 3′ provectors pNMDV503 (PlyCP26F) and pNMDV516 (PlyCP39O) and of assembled vectors pNMDV509 (psm), pNMDV600 (ZP173), pNMDV601 (ZP278), pNMDV599 (CP25L), are presented in Suppl. Fig. S7. Restriction and modification enzymes from Thermo Fisher Scientific Baltics (Vilnius, Lithuania) were used for all cloning steps.

### Lysin expression in plants

*Nicotiana benthamiana* plants were used as the expression host for these studies. The methods described generally translate to food species hosts, including *Beta vulgaris* (beet), *Spinacia oleracea* (spinach), or *Lactuca sativa* (lettuce). *N. benthamiana* plants were grown in a growth chamber at 25 °C with a 16 h light and 8 h dark photoperiod. Five to six-week-old plants were used for infiltration and for spraying with recombinant *A. tumefaciens*.

*A. tumefaciens* cultures were grown overnight at 30 °C in Lysogeny broth (LB) medium containing 50 mg/l rifampicin and other appropriate antibiotics depending on the type of plasmids (50 mg/l kanamycin for selection of integrase, TMV 3′ provector and TMV assembled vector; 50 mg/l carbenicillin for selection of 5′ TMV provectors). *Agrobacterium* overnight cultures were adjusted to an OD_600_ of 1.5, sedimented by centrifugation at 3220 × *g* for 5 min and suspended in tap water.

#### Syringe infiltration of *N. benthamiana* leaves

Plant agroinfiltration was performed as previously described^21^. A mix of three *A. tumefaciens* strains containing the 5′ provector, 3′ provector and integrase plasmid constructs was infiltrated into the abaxial side of the leaf using a syringe without a needle. MagnICON 5′ provector module for cytosol targeting (pICH20111) as well as the integrase expression vector pICH14011 were used together with 3´modules pNMDV503 and pNMDV516. Strains containing 5′ and 3′provector modules were used at dilution 1:100, and the integrase cassette - containing strain at dilution 1:40. Plant leaves were observed and collected at 5 to 6 days post infiltration. pICH20111 (5′ provector for cytosol expression) and pICH14011 (integrase) constructs are described in^32^.

#### Spraying of plant leaves

For lysin expression in *N. benthamiana* using assembled vectors, plant leaves were sprayed with a 1:1000 dilution of *A. tumefaciens* strain containing assembled vector as described in^33^. Spraying was performed with bacteria diluted in tap water and supplemented with 0.1% (v/v) Silwet L77 (Kurt Obermeier). Plant leaves were collected at 6 to 10 days post spraying.

### Preparation of crude protein extracts for bacteriolytic activity tests

Frozen plant material was homogenized with chilled mortar and pestle and mixed with extraction buffer (50 mM sodium phosphate, 5 mM DTT, 150 mM NaCl, pH 7.5) at a ratio of 1 g of tissue to 5 ml of buffer. Cell debris were removed by centrifugation at 21000 × g, at 4 °C for 30 min. The supernatant was considered as total soluble protein.

### Purification of lysins

Purification of lysins followed the general scheme: homogenization of sample, clarification, HIC chromatography, followed by desalting and CEXC or AEXC column. Detailed purification protocols of each lysin as well as SDS-PAGE gel pictures can be found in Suppl. Text S1 and Suppl. Fig. S2.

### Sequence verification of protein termini

Matrix-assisted laser desorption/ionization (MALDI) time-of-flight (TOF) mass spectrometry determination of *C. perfringens* bacteriophage lysins molecular mass and protein termini has been done as described in^34^ and in Supplementary Text S2.

### Bacteriolytic activity

#### Turbidity reduction assay and cfu counts

Crude protein extracts or purified lysins were used in bacteriolytic activity assays. *C. perfringens* cultures were grown in TSB under anaerobic conditions to OD_600_∼0.7–0.8, sedimented by centrifugation and suspended in a buffer of choice. Bacterial suspension was mixed with 1–50 µl of purified lysin or crude protein extract in 1 ml and incubated at RT. OD_600_ measurements were taken with spectrophotometer (Genesys 20, Thermo Scientific) every 5–10 minutes. Experiments were performed in triplicate.

Serial dilutions for cfu (colony forming units) enumeration were done in PBS, pH 7.3 after 60 min. co-incubation of bacteria with plant extracts. 30 µl of each sample was plated on TSA plates. Cfu were counted following overnight incubation under anaerobic conditions at 37 °C. 25 cfu per plate of undiluted sample was considered as lowest detection limit (833 cfu/ml or 2.9 log cfu/ml)^35^. All experiments were repeated 3 to 7 times.

#### Bacteriolytic activity in cooked meat

*C. perfringens* NCTC8237 was used as a model pathogen strain. Cultures were grown to OD_600_∼0.3 in TSB anaerobically, then diluted to OD_600_ = 0.025. Samples of 10 g of minced cooked turkey meat were combined with 3 ml of citrate-phosphate buffer supplemented with 856 mM NaCl, pH 5.5 and 100 µl of diluted bacteria (4 log cfu/ml). 25 µg of purified lysin or 50 µg of nisin was added, thoroughly mixed and incubated at RT anaerobically. Nisin (Sigma) 5 mg/ml stock was prepared by dissolving powder in 0.02 N HCl.

Serial dilutions of analysed samples were done in citrate-phosphate buffer supplemented with 256 mM NaCl, pH 5.5, and 50 µL of each sample were plated on TSA plates. Bacterial cfu/ml enumeration was done following overnight incubation under anaerobic conditions at 37 °C. A count of 25 cfu per plate of undiluted sample was considered the lower limit of detection (500 cfu/ml or 2.7 log_10 _cfu/ml)^35^.

#### Microscopy

*C. perfringens* cultures were grown in TSB under anaerobic conditions to OD_600_∼0.7-0.8, sedimented by centrifugation, suspended in citrate-phosphate buffer with 50 mM NaCl (pH 5.5) and treated with 5.5 µg/ml of nisin or with 5 µg/ml of ZP173. Microscopy images were taken after 90 min of incubation at RT with help of Olympus CKX41 Inverted Phase Contrast Microscope and QIMAGING MicroPublisher 3.3 RTV Color Cooled CCD.

## Electronic supplementary material

Supplementary information
