# Supplementary material for: Plant-expressed bacteriophage lysins control pathogenic strains of Clostridium perfringens
Source: Sci Rep. 2018 Jul 12;8:10589. doi: 10.1038/s41598-018-28838-4 (PMC6043497; doi:10.1038/s41598-018-28838-4)
Supplement: Supplementary file 1 — Supplementary information [file 41598_2018_28838_MOESM1_ESM.pdf]

Plant-expressed bacteriophage lysins control pathogenic strains of Clostridium perfringens

Vaiva Kazanavičiūtė, Audrius Misiūnas, Yuri Gleba, Anatoli Giritch, Aušra Ražanskienė

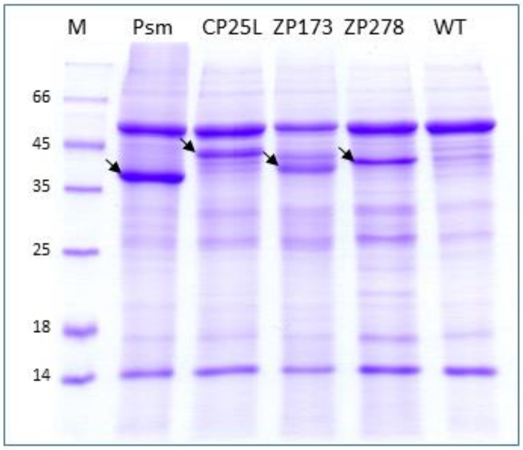

Supplementary Figure S1. Expression of *C. perfringens* bacteriophage lysins in spinach. Coomasie stained SDS-PAGE of crude plant extracts. Plant material (*S. oleracea* “Frühes Riesenblatt”) (50 mg) was harvested at 10 days post spraying, ground in liquid nitrogen and extracted with 50 mM sodium phosphate, 5 mM DTT, 150 mM NaCl, (pH 7.5). 3 µg of plant extract was resolved in 12.5% polyacrylamide gel for Coomassie staining. Mw – Unstained protein MW marker (Thermo Fisher Scientific Baltics), psm, CP25L, ZP173, ZP278 – extracts of *S. oleracea* leaves, transfected with chloroplast-targeted lysins expression constructs, Wt – crude extract of non - sprayed *S. oleracea* leaves. Bands corresponding to recombinant lysins are marked by arrows.

Supplementary Text S1. Purification of lysins.

ZP173.

A small portion of frozen leaf tissue was homogenized with chilled mortar and pestle in liquid nitrogen. The powder was mixed with cold extraction buffer (50 mM NaH_2PO_4/Na_2HPO_4, 2 mM DTT, pH 5.0) at a ratio of 1 g of plant material to 5 ml of buffer and kept on ice for 10-15 min. Cell debris were removed by centrifugation at 3220 *g,* at 4 °C for 20 min. Pellets were discarded and the supernatant was filtered by passing the solution through membrane filters (pore sizes 5 µm and 0.22 µm). The pH of solution was adjusted to 5 and formed precipitate was removed by centrifugation at 3220 *g,* at 4 °C for 5 min. The supernatant was taken as total soluble protein and applied for purification in two steps.

At the first purification step the chromatography column was filled with Butyl sepharose FF resin (GE Healthcare Life Sciences, Uppsala, Sweden) and pre-equilibrated with cold buffer (50 mM NaH_2PO_4/Na_2HPO_4, 1.1 M $(NH_4)_2SO_4$, 2 mM DTT, pH 7.0). Protein solution was loaded onto the column and the Butyl sepharose bounded protein fraction was eluted by washing with elution buffer (50 mM NaH_2PO_4/Na_2HPO_4, 0.77 M $(NH_4)_2SO_4$, 2 mM DTT, pH 7.0). Collected protein fraction was transferred to the diafiltrating concentrator (10 kDa) and centrifuged at 3220 *g* until the volume of protein solution decreased 8-10 folds. Concentrate was diluted up to a primary volume with buffer containing 50 mM NaH_2PO_4/Na_2HPO_4, 2 mM DTT (pH 5.0). Procedure was repeated till conductivity decreased below 9 mS/cm, and protein solution subjected to the final purification step using SP sepharose FF resin (GEHealthcare Life Sciences, Uppsala, Sweden). Chromatography media was pre-equilibrated with cold buffer (50 mM NaH_2PO_4/Na_2HPO_4, 2 mM DTT, pH 5.0). Protein solution was loaded onto the column and SP sepharose bounded protein fraction was eluted by linear gradient of cold washing buffer supplemented with 300 mM of NaCl. Collected ZP173 was freeze-dried.

ZP278.

A small portion of frozen leaf tissue was homogenized with chilled mortar and pestle in liquid nitrogen. The powder was mixed with cold extraction buffer (50 mM NaH_2PO_4/Na_2HPO_4, 150

mM NaCl, 5 mM DTT, pH 7.0) at a ratio of 1 g of plant material to 5 ml of buffer and kept on ice for 10-15 min. Cell debris were removed by centrifugation at 3220 *g,* at 4 °C for 20 min. Pellets were discarded and the supernatant was filtered by passing solution through membrane filters (pore sizes 5 µm and 0.22 µm). The pH of solution was adjusted to 6.5 and formed precipitate was removed by centrifugation at 3220 *g,* at 4 °C for 5 min. The supernatant was taken as total soluble protein and applied for purification in two steps.

At the first purification step the chromatography column was filled with Butyl sepharose FF resin (GE Healthcare Life Sciences, Uppsala, Sweden) and pre-equilibrated with cold buffer (50 mM NaH_2PO_4/Na_2HPO_4, 1.2 M $(NH_4)_2SO_4$, 5 mM DTT, pH 6.5). Protein solution was loaded onto the column and the Butyl sepharose bounded protein fraction was eluted by washing with elution buffer (50 mM NaH_2PO_4/Na_2HPO_4, 0.78 M $(NH_4)_2SO_4$, 5 mM DTT, pH 6.5). Collected protein fraction was transferred to the diafiltrating concentrator (10 kDa) and centrifuged at 3220 *g* until the volume of protein solution decreased 8-10 folds. Concentrate was diluted up to a primary volume with buffer containing 50 mM NaH_2PO_4/Na_2HPO_4, 5 mM DTT (pH 7.0). Procedure was repeated till conductivity decreased below 9 mS/cm, and protein solution was subjected to the final purification step using DEAE sepharose FF resin (GEHealthcare Life Sciences, Uppsala, Sweden). Chromatography media was pre-equilibrated with cold buffer (50 mM NaH_2PO_4/Na_2HPO_4, 5 mM DTT, pH 7.0). Protein solution was loaded onto the column and DEAE sepharose bounded protein fraction was eluted by linear gradient of cold washing buffer additionally containing 250 mM of NaCl. Collected ZP278 was freeze-dried.

CP25L.

A small portion of frozen leaf tissue was homogenized with chilled mortar and pestle in liquid nitrogen. Prepared powder was mixed with cold extraction buffer (50 mM NaH_2PO_4/Na_2HPO_4, 100 mM NaCl, 2 mM DTT, pH 7.5) at a ratio of 1 g of plant material to 5 ml of buffer. The crude extract was kept on ice for 10-15 min. Cell debris were removed by centrifugation at 3220 *g,* at 4 °C for 20 min. Pellets were discarded and the supernatant was filtered by passing solution through membrane filters (pore sizes 5 µm and 0.22 µm). The pH of solution was adjusted to

6.5 and formed precipitate was removed by centrifugation at 3220 *g,* at 4 °C for 5 min. The supernatant was taken as total soluble protein and applied for purification in two steps.

At the first purification step the chromatography column was filled with Butyl sepharose FF resin (GE Healthcare Life Sciences, Uppsala, Sweden) and pre-equilibrated with cold buffer (50 mM NaH_2PO_4/Na_2HPO_4, 0.85 M $(NH_4)_2SO_4$, 2 mM DTT, pH 6.5). Protein solution was loaded onto the column and the Butyl sepharose bounded protein fraction was eluted by washing with elution buffer (50 mM NaH_2PO_4/Na_2HPO_4, 0.6 M $(NH_4)_2SO_4$, 2 mM DTT, pH 6.5). Collected protein fraction was transferred to the diafiltrating concentrator (10 kDa) and centrifuged at 3220 *g* until the volume of protein solution decreased 6-8 folds. Concentrate was diluted up to a primary volume with buffer containing 20 mM NaH_2PO_4/Na_2HPO_4, 2 mM DTT (pH 8). Procedure was repeated till conductivity decreased below 5 mS/cm and protein solution was subjected to the final purification step using Q sepharose FF resin (GEHealthcare Life Sciences, Uppsala, Sweden). Chromatography media was pre-equilibrated with cold buffer (20 mM NaH_2PO_4/Na_2HPO_4, 2 mM DTT, pH 8.0). Protein solution was loaded onto the column and Q sepharose bounded protein fraction was eluted by linear gradient of cold washing buffer supplemented with 125 mM of NaCl. Collected CP25L was freeze-dried.

PlyCP26F.

A small portion of frozen leaf tissue was homogenized with chilled mortar and pestle in liquid nitrogen. Prepared powder was mixed with cold extraction buffer (50 mM NaH_2PO_4/Na_2HPO_4, 150 mM NaCl, 2 mM DTT, pH 7.5) at a ratio of 1 g of plant material to 5 ml of buffer. The crude extract kept on ice for 10-15 min. Cell debris were removed by centrifugation at 3220 *g,* at 4 °C for 20 min. Pellets were discarded and the supernatant was filtered by passing solution through membrane filters (pore sizes 5 µm and 0.22 µm). The pH of solution was adjusted to 7.0 and formed precipitate was removed by centrifugation at 3220 *g,* at 4 °C for 5 min. The supernatant was taken as total soluble protein and applied for purification in two steps.

At the first purification step the chromatography column was filled with Butyl sepharose FF resin (GE Healthcare Life Sciences, Uppsala, Sweden) and pre-equilibrated with cold buffer (50 mM NaH_2PO_4/Na_2HPO_4, 1.2 M $(NH_4)_2SO_4$, 2 mM DTT, pH 7.0). Protein solution was loaded onto

the column and the Butyl sepharose bounded protein fraction was eluted by washing with elution buffer (50 mM NaH_2PO_4/Na_2HPO_4, 0.84 M $(NH_4)_2SO_4$, 2 mM DTT, pH 7.0). Collected protein fraction was transferred to the diafiltrating concentrator (10 kDa) and centrifuged at 3220 *g* until the volume of protein solution decreased 8-10 folds. Concentrate was diluted up to a primary volume with buffer containing 50 mM NaH_2PO_4/Na_2HPO_4, 2 mM DTT (pH 6). Procedure was repeated till conductivity decreased below 7 mS/cm and protein solution subjected to the final purification step using SP sepharose FF resin (GEHealthcare Life Sciences, Uppsala, Sweden). Chromatography media was pre-equilibrated with cold buffer (50 mM NaH_2PO_4/Na_2HPO_4, 2 mM DTT, pH 6.0). Protein solution was loaded onto the column and SP sepharose bounded protein fraction was eluted with elution buffer (50 mM NaH_2PO_4/Na_2HPO_4, 1.0 M NaCl, 2 mM DTT, pH 6.0). Collected PlyCP26F was freeze-dried.

PlyCP39O.

A small portion of frozen leaf tissue was homogenized with chilled mortar and pestle in liquid nitrogen. Prepared powder was mixed with cold extraction buffer (50 mM NaH_2PO_4/Na_2HPO_4, 200 mM NaCl, 5 mM DTT, pH 7.5) at a ratio of 1 g of plant material to 5 ml of buffer. The crude extract was kept on ice for 10-15 min. Cell debris were removed by centrifugation at 3220 *g,* at 4 °C for 20 min. Pellets were discarded and the supernatant was filtered by passing solution through membrane filters (pore sizes 5 µm and 0.22 µm). The pH of solution was adjusted to 7.0 and formed precipitate was removed by centrifugation at 3220 *g,* at 4 °C for 5 min. The supernatant was taken as total soluble protein and applied for purification in two steps.

At the first purification step the chromatography column was filled with Butyl sepharose FF resin (GE Healthcare Life Sciences, Uppsala, Sweden) and pre-equilibrated with cold buffer (50 mM NaH_2PO_4/Na_2HPO_4, 1.2 M $(NH_4)_2SO_4$, 5 mM DTT, pH 7.0). Protein solution was loaded onto the column and the Butyl sepharose bounded protein fraction was eluted by washing with elution buffer (50 mM NaH_2PO_4/Na_2HPO_4, 0.78 M $(NH_4)_2SO_4$, 5 mM DTT, pH 7.0). Collected protein fraction was transferred to the diafiltrating concentrator (10 kDa) and centrifuged at 3220 *g* until the volume of protein solution decreased 6-8 folds. Concentrate was diluted up to a primary volume with buffer containing 50 mM NaH_2PO_4/Na_2HPO_4, 5 mM DTT (pH 6).

Procedure was repeated till conductivity decreased below 17 mS/cm and protein solution was subjected to the final purification step using SP sepharose FF resin (GEHealthcare Life Sciences, Uppsala, Sweden). Chromatography media was pre-equilibrated with cold buffer (50 mM NaH_2PO_4/Na_2HPO_4, 120 mM NaCl, 5 mM DTT, pH 7.0). Protein solution was loaded onto the column and SP sepharose bounded protein fraction was eluted by linear gradient of cold buffer containing 350 mM of NaCl. Collected PlyCP39O was freeze-dried.

psm.

A small portion of frozen leaf tissue was homogenized with chilled mortar and pestle in liquid nitrogen. Prepared powder was mixed with cold extraction buffer (50 mM NaH_2PO_4/Na_2HPO_4, 150 mM NaCl, 2 mM DTT, pH 5.0) at a ratio of 1 g of plant material to 5 ml of buffer. The crude extract was kept on ice for 10-15 min. Cell debris were removed by centrifugation at 3220 *g,* at 4 °C for 20 min. Pellets were discarded and the supernatant was filtered by passing solution through membrane filters (pore sizes 5 µm and 0.22 µm). The pH of solution was adjusted to 6.0 and formed precipitate was removed by centrifugation at 3220 *g,* at 4 °C for 5 min. The supernatant was taken as total soluble protein and applied for purification in two steps.

At the first purification step the chromatography column was filled with Butyl sepharose FF resin (GE Healthcare Life Sciences, Uppsala, Sweden) and pre-equilibrated with cold buffer (50 mM NaH_2PO_4/Na_2HPO_4, 1.2 M $(NH_4)_2SO_4$, 2 mM DTT, pH 6.0). Protein solution was loaded onto the column and the Butyl sepharose bounded protein fraction was eluted by washing with elution buffer (50 mM NaH_2PO_4/Na_2HPO_4, 0.78 M $(NH_4)_2SO_4$, 2 mM DTT, pH 6.0). Collected protein fraction was transferred to the diafiltrating concentrator (10 kDa) and centrifuged at 3220 *g* until the volume of protein solution decreased 8-10 folds. Concentrate was diluted up to a primary volume with buffer containing 50 mM NaH_2PO_4/Na_2HPO_4, 2 mM DTT (pH 8). Procedure was repeated till conductivity decreased below 8 mS/cm and protein solution was subjected to the final purification step using Q sepharose FF resin (GEHealthcare Life Sciences, Uppsala, Sweden). Chromatography media was pre-equilibrated with cold buffer (50 mM NaH_2PO_4/Na_2HPO_4, 2 mM DTT, pH 8.0). Protein solution was loaded onto the column and Q

sepharose bounded protein fraction was eluted by linear gradient of cold washing buffer supplemented with 250 mM of NaCl. Collected psm was freeze-dried.

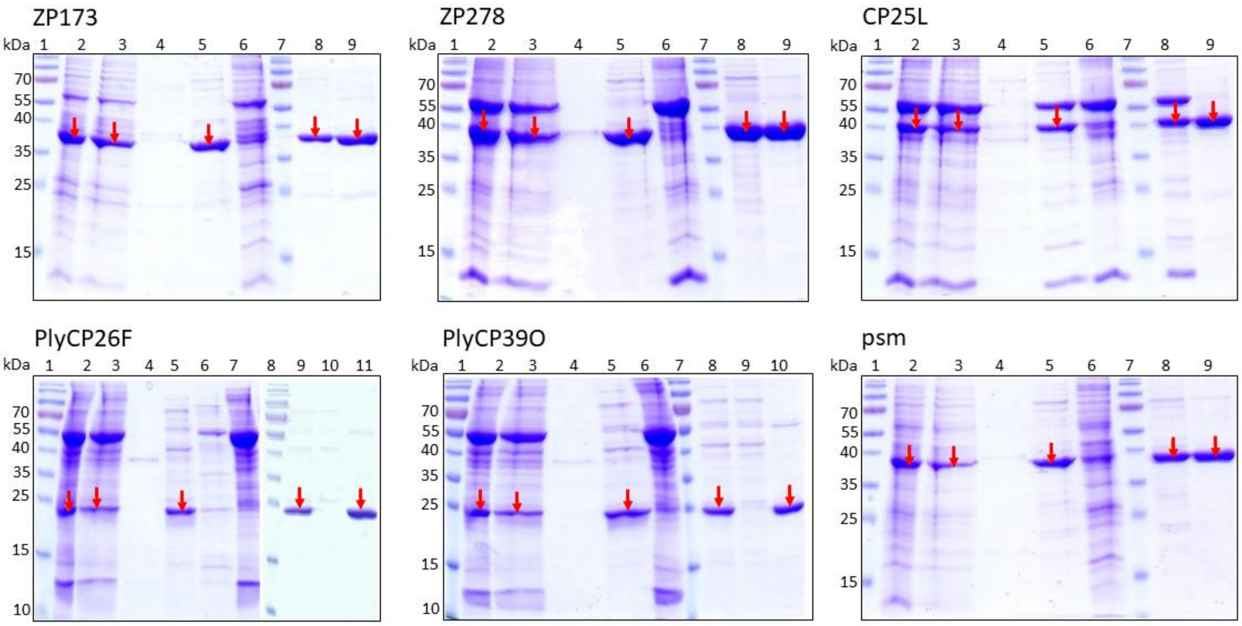

Supplementary Figure S2. Purification of plant – produced lysins. ZP173 – lane 1 and 7 - PageRuler™ Prestained Protein Ladder, lane 2 – extracted proteins, lane 3 – loaded proteins on Butyl sepharose, lane 4 – flow-through Butyl sepharose, lane 5 – collected proteins after Butyl sepharose I, lane 6 – collected proteins after Butyl sepharose II, lane 8 - loaded proteins on SP sepharose, lane 9 – collected proteins after SP sepharose. **ZP278** - lane 1 and 7 - PageRuler™ Prestained Protein Ladder, lanes 2 – extracted proteins, lane 3 - loaded proteins on Butyl sepharose, lane 4 – flow through Butyl sepharose, lane 5 – collected proteins after Butyl sepharose I, lane 6 – collected proteins after Butyl sepharose II, lane 8 – loaded proteins on DEAE sepharose, lane 9 – collected proteins after DEAE sepharose. **CP25L** – lane 1 and 7 - PageRuler™ Prestained protein ladder, lane 2 – extracted proteins, lane 3 – loaded proteins on Butyl sepharose, lane 4 – flow through Butyl sepharose, lane 5 – collected proteins after Butyl sepharose I, lane 6 – collected proteins after Butyl sepharose II, lane 8 – loaded proteins on Q sepharose, lane 9 – collected proteins after Q sepharose. **PlyCP26F** – lane 1 and 8 - PageRuler™ Prestained protein ladder, lane 2 – extracted proteins, lane 3 – loaded proteins on Butyl sepharose, lane 4 – flow through Butyl sepharose, lane 5 – collected proteins after Butyl sepharose I, lane 6 – collected proteins after Butyl sepharose II, lane 7 – collected proteins after Butyl sepharose III, lane 9 – loaded proteins on SP sepharose, lane 10 – flow through SP sepharose, lane 11 – collected proteins after SP sepharose. **PlyCP39O** – lane 1 and 7- PageRuler™ Prestained protein ladder**,** lane 2 – extracted proteins**,** lane 3 – loaded proteins on Butyl sepharose**,** lane 4 – flow through Butyl sepharose**,** lane 5 – collected proteins after Butyl sepharose I, lane 6 – collected proteins after Butyl sepharose II, lane 8 - loaded proteins on SP sepharose, lane 9 – flow through SP sepharose, lane 10 – collected proteins after SP sepharose. **psm** – lane 1 and 7- protein ladder**,** lane 2 – extracted proteins**,** lane 3 – loaded proteins on Butyl sepharose**,** lane 4 – flow through Butyl sepharose**,** lane 5 – collected proteins after Butyl sepharose I, lane 6 – collected proteins after Butyl sepharose II, lane 8 - loaded proteins on Q sepharose, lane 9 – collected proteins after Q sepharose.

Supplementary Text S2. MALDI-MS determination of *C. perfringens* bacteriophage lysins molecular mass and protein termini

Methods

Host plants were inoculated with *Agrobacterium* vectors carrying inserts for individual lysins. The gene sequences for lysin inserts in the expression vectors were verified by DNA sequencing. Lysins were extracted from plants and purified as described; each purified lysin was run on SDS-PAGE gels and stained with Coomassie for visualization or lyophilized and stored at -20 °C until further processing.

The composition of the plant-expressed proteins was verified by complementary mass spectrometry methods, including in-source decay (ISD) and T3-sequencing by Fraunhofer Institute for Cell Therapy and Immunology IZI, Department of Drug Design and Target Validation, Halle (Saale), Germany:

Molecular mass analysis. MALDI-TOF/TOF mass spectrometer was calibrated using mass signals of a set of standard peptides and proteins with known masses. Spectra employed for calibration were acquired with the same laser energy as used for sample analysis;

Sequence verification of the protein termini. Specialized mass spectrometry technique termed in-source decay (ISD) was used. This technique makes use of N-terminal and C-terminal fragment ions, which are generated due to highly elevated laser energy levels during ionization. These fragment ions can be used to derive the terminal amino acid sequences of proteins. ISD spectra do not directly cover the first amino acids of the N- and C- terminus and hence, do often not allow the unambiguous identification/confirmation of the respective amino acids as well as the exact localization of possible modifications. To solve this issue, a technique termed as T3-sequencing is used. The T3 approach is based on the analysis of selected ISD fragments by LIFT. Since ISD fragment ions are generated within the ion source, they can further fragment inside the mass analyzer. LIFT specifically selects an ISD fragment ion and acquires a fragment (MS/MS) spectrum of it. This fragment spectrum usually allows the direct identification of the first amino acids and their modifications.

Results

When combined, the methods described above provide sufficient data to ensure that all plant produced lysins are intact, and no truncation of N- and C-terminal ends is detected. Three of analyzed lysins (psm, ZP173 and ZP278) have acetylated N-termini. Determined molecular masses of all lysins match theoretical values suggesting that there are no modifications in amino acid sequences of plant-produced lysins. Suppl. Fig 3 presents MALDI-TOF mass spectrum acquired from ZP278 as a representative lysin. Suppl. Fig 4 presents in-source decay analysis of ZP173. Suppl. Fig 5A and B presents T3-sequencing analysis of ZP278. All lysins were analyzed using the same methods.

Supplementary Table S1. Summary of MS analyses results for 4 bacteriophage lysins. The table shows the results of analysis of plant-made lysins and published results of analyses of bacterial proteins, where available. Post-translational processing of the plant-made polypeptides, if any, is also shown.

Lysin	Plant-made (determined by Notifier)			Bacterial (literature values)
	N-terminus	C-terminus	Determined molecular mass (vs theoretical)	N-terminus (method). Reference
psm	Confirmed. N-terminal met is present. N-terminus is acetylated.	Confirmed	38648.0 (38635.7)	N- terminal met is present in *E. coli* expressed protein. (X-ray structure data) [1]
ZP173	Confirmed. N-terminal met is present. N-terminus is acetylated.	Confirmed	38652.9 (38660.1)	No data available
ZP278	Confirmed. N-terminal met is present. N-terminus is acetylated.	Confirmed	40222.4 (40208.4)	No data available
CP25L	Confirmed. N-terminal Met is present.	Confirmed	43418.0 (43400.9)	No data available

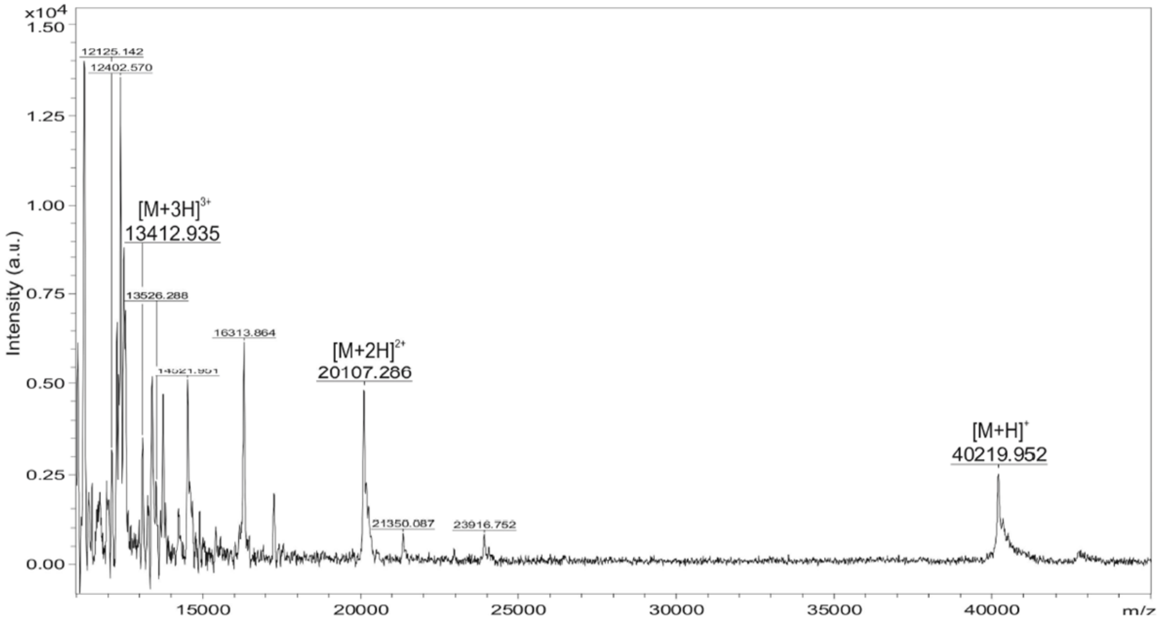

Supplementary Figure S3. MALDI-TOF mass spectrum acquired from ZP278. The acquired MALDI-TOF mass spectrum displayed mass signals for the single and the multiple charged molecular ion of ZP278. Further mass signals that could belong to truncated or modified ZP278 were not detected, indicating that only one proteoform was present. The determined molecular mass displayed a deviation of +56.0 Da compared with the theoretical value, which points towards the presence of an acetylation.

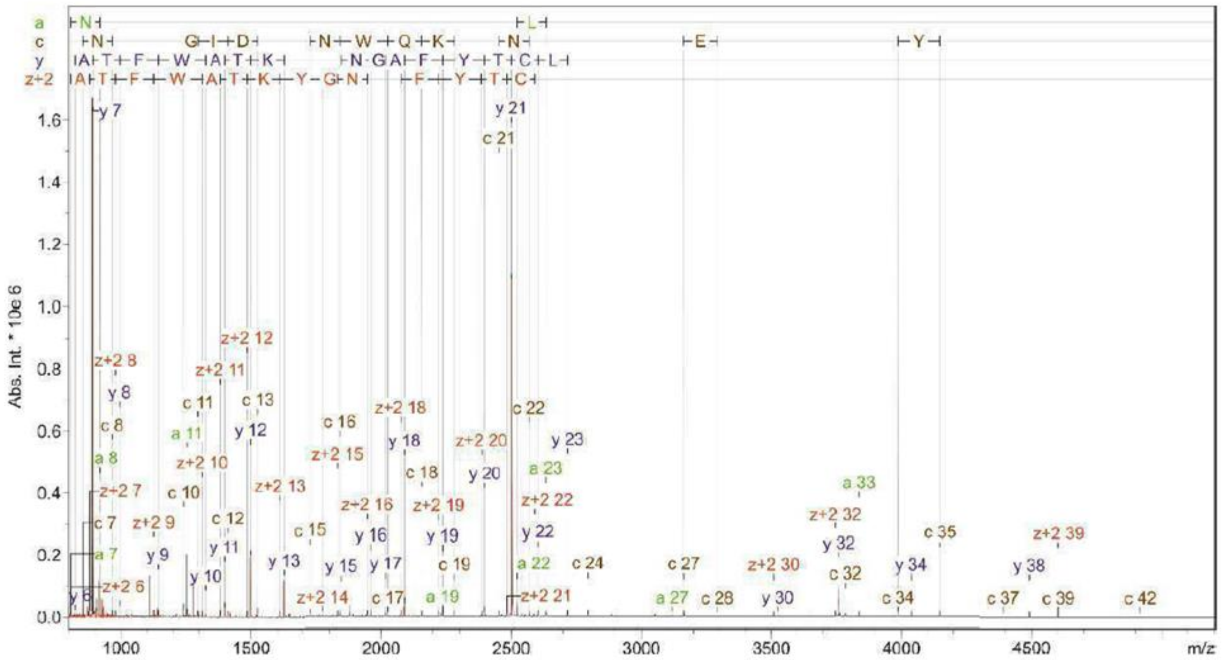

Supplementary Figure S4. In-source decay analysis of ZP178. ISD analysis of ZP278 delivered a fragment spectrum with many ISD fragment signals. Sequence information for the N- and C- terminus of ZP278 was obtained for the a-, c-, y- and z+2-type ion series. These ion series indicate that neither the N- nor C-terminus were truncated and that the N-terminus was acetylated. Further modifications were not detected.

A

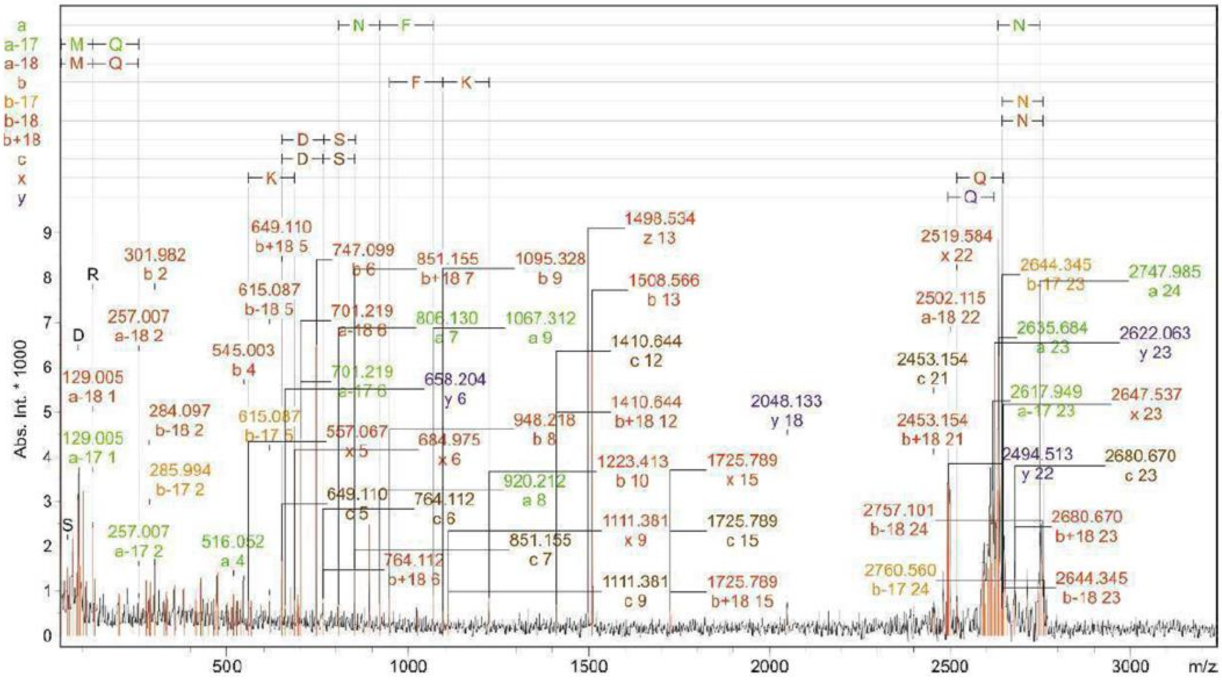

B

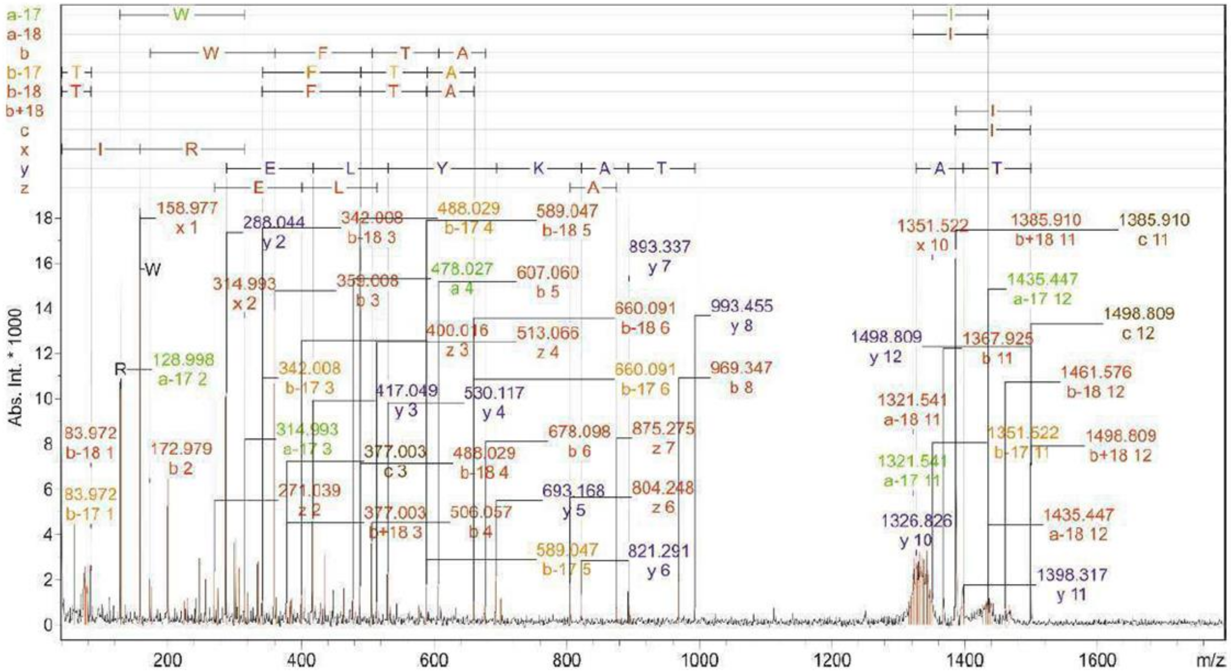

Supplementary Figure S5. MS/MS spectrum acquired from ZP278 ISD fragment ion C_{24} (A) and ion y_{12} (B). The MS/MS spectrum acquired for c_{24} indicates that acetylation is present at the N-terminus. The MS/MS spectrum acquired for y_{12} points towards an intact C-terminus without modifications.

Supplementary Table S2. *C. perfringens* strains used in the study.

No.	Strain	Isolated from	Characteristics	Reference if possible
1	NCTC2837	-	Type A. a-toxin gene positive.	-
2	NCTC6785 (ATCC 10873)	-	Type A. a-toxin gene positive.	[2]
3	NCTC8235 (ATCC 12922)	Stew	Type A, cpa and cpe genes present.	[3]
4	NCTC8237 (ATCC 13124)	-	Type A, a-toxigenic, cpa and pfoA genes present.<br>Type strain.	[4]
5	NCTC8238 (ATCC 12916)	Boiled salt beef	Type A. The presence of cpa and cpe genes was confirmed by PCR.	[3]
6	NCTC8239 (ATCC 12917)	Boiled salt beef	NCTC: Type A. a-toxin gene positive. Contains a fragment of the enterotoxin gene.<br>ATCC: Type D. Heat resistance of spores<br>Agglutinating type 3. The presence of cpa, etx, and cpe genes was confirmed by PCR.	[3]
7	NCTC8247 (ATCC 12918)	Faeces	Type A. a-toxin gene positive. This isolate also contains a fragment of the enterotoxin gene	[3]
8	NCTC8359 (ATCC 12915)	Stewed steak	Type A. The presence of cpa and cpe genes was confirmed by PCR.	[3]
9	NCTC8449 (ATCC 12921)	Steamed lamb	Type A. a-toxin gene positive. This isolate also contains a fragment of the enterotoxin gene	[3]
10	NCTC8678 (ATCC 12919)	Human faeces, food poisoning	NCTC: Type A, Epsilon toxin –ve<br>Agglutinating type 5<br>ATCC: Type D. The presence of cpa, etx, and cpe genes was confirmed by PCR.	[3]
11	NCTC8679 (ATCC 12920)	Human faeces, food poisoning	Type A. a-toxin gene positive. Contains a fragment of the enterotoxin gene.	[3]

12	NCTC8797	Salt beef	Type A. a-toxin gene positive. This isolate also contains a fragment of the enterotoxin gene	[3]
13	NCTC8798	Meat rissole, food poisoning outbreak in a school	Type A. a-toxin gene positive. This isolate also contains a fragment of the enterotoxin gene	[3]
14	NCTC9851 (ATCC 12925)	Braised heart	Type A. Agglutinating type 11 The presence of cpa and cpe genes was confirmed by PCR.	[3]
15	NCTC10239 (ATCC 14809)	Rissoles	NCTC: Type A. a-toxin gene positive. This isolate also contains a fragment of the enterotoxin gene<br>ATCC:Type D. The presence of cpa, etx, and cpe genes was confirmed by PCR.	[3]
16	NCTC10240 (ATCC 1481)	Chicken	Type A. a-toxin gene positive. This isolate also contains a fragment of the enterotoxin gene	[3]
17	NCTC10611	-	Type A	[3]
18	NCTC10612	Human faeces (food poisoning outbreak)	Type A	[3]
19	NCTC10613	Minced beef, food poisoning	Type A.	-
20	NCTC10614	Human faeces, food poisoning case	Type A	[3]
21	NCTC11144	Beef (food poisoning outbreak)	Type A.	[3]
22	DSM11779	Boulette (Hamburger)	Type A, betahaemolytic	-
23	DSM11780	Human faeces	Type A, betahaemolytic	-
24	DSM11781	Boulette (Hamburger)	Type A, betahaemolytic	-
25	DSM11782	Human faeces	Type A, betahaemolytic	-
26	DSM11783	Human faeces	Type A, non-haemolytic	-

Supplementary Table S3. *A. tumefaciens* strains used in this study

Strains	Description	References
A. tumefaciens GV3101	(pMP90RK), nopaline, Rif^r	4
GV3101(pICH20111)	Contains TMV virus-based 5' provector for cytosolic expression	Icon Genetics
GV3101(pICH1401)	Contains integrase expression cassette	Icon Genetics
GV3101(pNMDV503)	Contains TMV virus-based 3' provector with PlyCP26F	This study
GV3101(pNMDV516)	Contains TMV virus-based 3' provector with PlyCP39O	This study
GV3101(pNMDV509)	Contains TMV virus-based vector for cytosol expressed psm	This study
GV3101(pNMDV600)	Contains TMV virus-based vector for cytosol expressed ZP173	This study
GV3101(pNMDV599)	Contains TMV virus-based vector for cytosol expressed CP25L	This study
GV3101(pNMDV601)	Contains TMV virus-based vector for cytosol expressed ZP278	This study

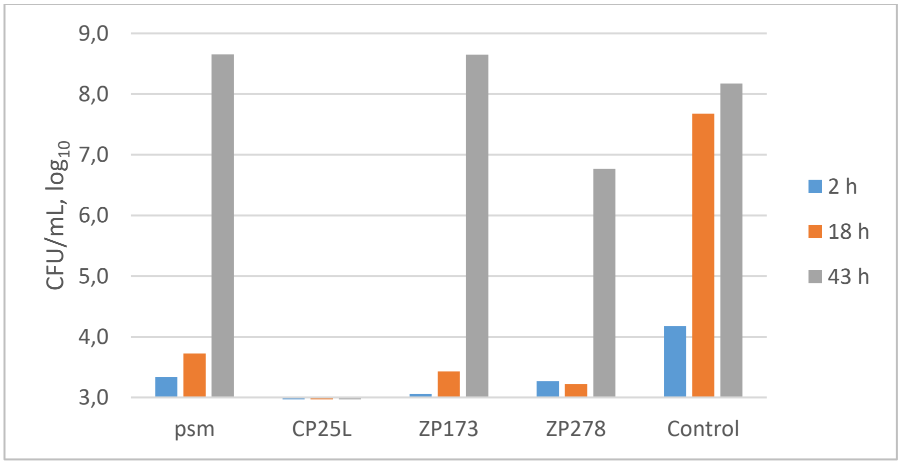

Supplementary Figure S6. Activity of purified lysins against a mix of 5 food-related *C. perfringens* strains in cooked turkey meat. *C. perfringens* strains NCTC8235, NCTC8239, NCTC9851, NCTC8449 and NCTC8797 were grown to OD_{600} appr. 0.23 in TSB anaerobically. Each strain was diluted to OD600 =0.005 and mixed in equal amounts to get 1 ml of bacterial culture

of OD600=0.025. 10 g cooked turkey breast meat was combined with 100 µl of mixed diluted bacterial culture, 3 ml citrate-phosphate buffer with 856 mM NaCl, pH 5,5, 50 µg purified lysin or nisin and incubated at RT anaerobically. 2 h, 18 h and 43 h – cfu counts of samples, incubated at RT anaerobically for the indicated time.

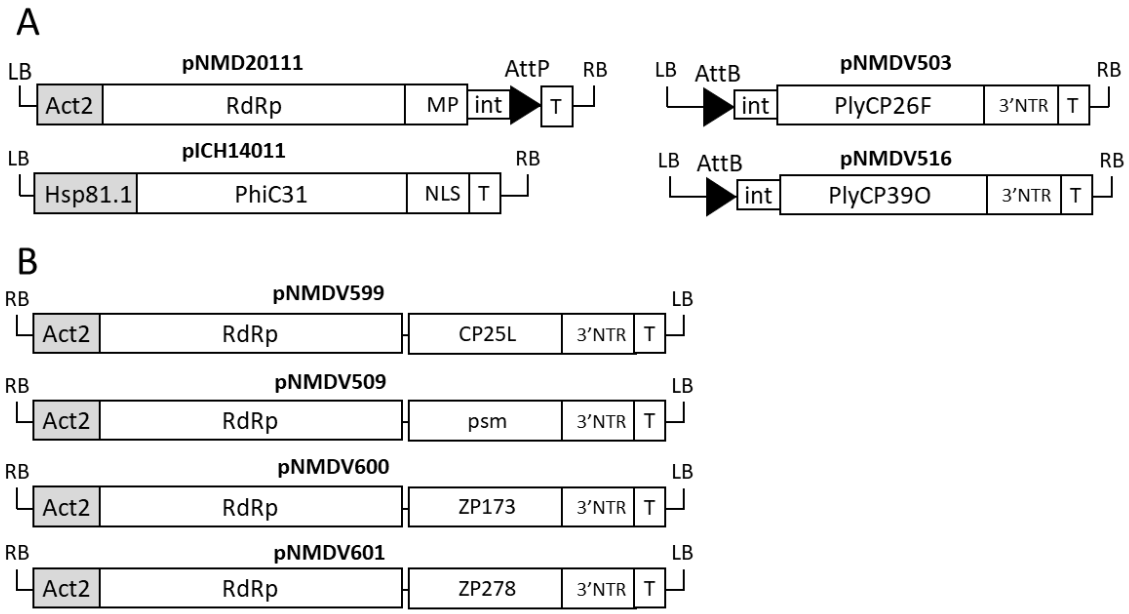

Supplementary Figure 7. **Schematic representation of T-DNA regions of used TMV-based provector modules (A) and assembled vectors (B).** LB – left T-DNA border, RB – right T-DNA border, Act2 and Hsp81.1 –promoters, RdRp –RNA-dependent RNA polymerase, MP –TMV movement protein, int – intron, T – nos terminator, AttP and AttB - integrase recombination sites, PhiC31 - *Streptomyces* phage C31 integrase, NLS - nuclear localization signal.

REFERENCES

1 Tamai, E. *et al.* X-ray structure of a novel endolysin encoded by episomal phage phiSM101 of Clostridium perfringens. *Mol Microbiol* **92**, 326-337, doi:10.1111/mmi.12559 (2014).

2 Epps, H. M. Studies on bacterial amino-acid decarboxylases: 4. l(-)-histidine decarboxylase from Cl. welchii Type A. *Biochem J* **39**, 42-46 (1945).

3 Hobbs, B. C., Smith, M. E., Oakley, C. L., Warrack, G. H. & Cruickshank, J. C. Clostridium welchii food poisoning. *J Hyg (Lond)* **51**, 75-101 (1953).
4 Koncz, C., Schell, J. The promoter of TL-DNA gene 5 controls the tissue-specific expression of chimaeric genes carried by a novel type of Agrobacterium binary vector. *Molec Gen Genet* **204**, doi:https://doi.org/10.1007/BF00331014 (1986).
